# Flavonone 3-hydroxylase Relieves Bacterial Leaf Blight Stress in Rice via Overaccumulation of Antioxidant Flavonoids and Induction of Defense Genes and Hormones

**DOI:** 10.3390/ijms22116152

**Published:** 2021-06-07

**Authors:** Rahmatullah Jan, Muhammad Aaqil Khan, Sajjad Asaf, Lubna, Jae-Ryoung Park, In-Jung Lee, Kyung-Min Kim

**Affiliations:** 1 Division of Plant Biosciences, Department of Applied Biosciences, College of Agriculture and Life Science, Kyungpook National University, 80 Dahak-ro, Buk-gu, Daegu 41566, Korea; rehmatbot@yahoo.com (R.J.); aqil_bacha@yahoo.com (M.A.K.); icd92@naver.com (J.-R.P.); ijlee@knu.ac.kr (I.-J.L.); 2 Costal Agriculture Research Institute, Kyungpook National University, 80 Dahak-ro, Buk-gu, Daegu 41566, Korea; 3 Natural and Medical Science Research Center, University of Nizwa, Nizwa 616, Oman; sajadasif2000@gmail.com; 4 Department of Botany, Garden Campus, Abdul Wali Khan University, Marda 23200, Pakistan; lubnabilal68@gmail.com

**Keywords:** bacterial leaf blight, kaempferol, antioxidant, malondialdehyde, antagonistic

## Abstract

Efficient accumulation of flavonoids is important for increased tolerance to biotic stress. Although several plant defense mechanisms are known, the roles of many pathways, proteins, and secondary metabolites in stress tolerance are unknown. We generated a flavanone 3-hydroxylase (F3H) overexpressor rice line and inoculated *Xanthomonas Oryzae* pv. *oryzae* and compared the control and wildtype inoculated plants. In addition to promoting plant growth and developmental maintenance, the overexpression of F3H increased the accumulation of flavonoids and increased tolerance to bacterial leaf blight (BLB) stress. Moreover, leaf lesion length was higher in the infected wildtype plants compared with infected transgenics. Kaempferol and quercetin, which scavenge reactive oxygen species, overaccumulated in transgenic lines compared with wildtypes in response to pathogenic infection, detected by scanning electron microscopy and spectrophotometry. The induction of F3H altered the antioxidant system and reduced the levels of glutathione peroxidase activity and malondialdehyde (MDA) contents in the transgenic lines compared with the wildtypes. Downstream gene regulation analysis showed that the expression of F3H increased the regulation of flavonol synthase (FLS), dihydroflavonol 4-reductase (DFR), and slender rice mutant (SLR1) during BLB stress. The analysis of SA and JA signaling revealed an antagonistic interaction between both hormones and that F3H induction significantly promoted SA and inhibited JA accumulation in the transgenic lines. SA-dependent nonexpressor pathogenesis-related (NPR1) and Xa1 showed significant upregulation in the infected transgenic lines compared with the infected control and wildtype lines. Thus, the overexpression of F3H was essential for increasing BLB stress tolerance.

## 1. Introduction

*Oryza sativa* rice is a staple food of a majority of the global population and its demand has increased, mostly in developing countries. Rice cultivation is limited by many factors, including land availability, soil conditions, water supply, quality of seeds, pests, weeds, pathogenic diseases, and other biological agents. Bacterial leaf blight (BLB) is a devastating disease in rice caused by *Xanthomonas Oryzae* pv. *oryzae* (*Xoo*) [1]. BLB can occur at any developmental stage and manifests as either leaf blight or welting of young plants (Kresek). To date, no chemical control for BLB is known. Several strategies have been used to avoid BLB epidemics and reduce yield loss, but due to variations in the sensitivity of pathogenic races, chemical applications have not been successful [2]. The development of resistant cultivars is more effective and environmentally friendly. Thus far, 46 genes resistant to BLB were found in rice, with most evaluated in rice breeding. Among these, Xa1, Xa7, and signaling pathways were found. Among PSMs, flavonoids are specialized metabolites because they synthesize in a species-specific manner [3]. Based on the structure, nine subclasses of flavonoids, namely, chalcones, flavones, flavonols, dihydroflavonols, flavandiols, anthocyanins, proanthocyanidins, isoflavonoids, and aurones 9, have been identified in terrestrial plants [4]. Flavonoids are functionally involved in physiological development and the plant’s response, where Xa21 provides broadspectrum tolerance to more than six races of BLB [5,6,7].

Plant secondary metabolites (PSMs) perform various functions in mediating the interaction between plants and other organisms. Various constitutive and pathogen-induced phytochemicals provide plant’s with innate immunity [8]. Studies on model plants, such as Arabidopsis (*Arabidopsis thaliana*), have advanced our understanding of the molecular mechanisms of pathogen-induced accumulation of defensive PSM. However, little is known about the mechanism of PSM-mediated plant immunity. Pathogenic microbial infection activates various defense responses against multiple biotic and abiotic stresses, where the responses include pigmentation, pollen tube development, UV light stress, pathogen infection, and herbivory [9]. Flavanone 3-hydroxylase (F3H) is one of the main facilitator genes in flavonoid biosynthesis in the PAL pathway. Recently F3H was elucidated as the modulator of brown plant hopper resistance [10]. Kaempferol (Kr), quercetin (Qu), and anthocyanin are important flavonoids that can be glycosylated into glycosidic derivatives by the addition of glucose at carbons 3 and 7 [11]. The glycosides of Kr and Qu are also flavonoids. They act as antioxidants, their hydroxyl group scavenges free radicals by donating an electron or hydrogen [12]. Kr and Qu are better antioxidants than other flavonoids [13]. BLB is a prominent cause of stress that can increase reactive oxygen species (ROS); a higher accumulation of flavonoids is influential for ROS scavenging [14]. Similar to other flavonoids, anthocyanin acts as a stress-reducing agent and interest in understanding the mechanism by which anthocyanin reduces plant stress is growing. Anthocyanin helps to cope with stress via ROS quenching and stress and hormonal signaling [14,15]. Studies have indicated that *F3H* expression increases the accumulation of flavonoids, namely, Kr, Qu, and anthocyanin, which alters the interaction between JA, GA, SLR1, and DELLA in response to stress.

Plants accumulate signaling molecules, such as SA and JA, which induce pathogenesis-related genes and antimicrobial metabolites. The mutual antagonism of SA and JA allows a plant to efficiently modulate defense responses against the pathogen and to selectively induce the defense machinery without stimulating inappropriate and possibly counterproductive responses [16]. Although the exact point of mutualism is unknown, evidence suggests that SA-dependent signaling downregulates JA signaling in an NPR1-dependent manner [17]. However, studies have reported that mutualism depends on JIN1 and Col1 [18]. Similarly, the JIN1 mutant increases the accumulation of SA, indicating that JA interferes with SA synthesis [18]. NPR1, WRKY70, ERF1, and JAZ1-JAZ3 were shown to be involved in the SA–JA antagonism in Arabidopsis [19]. However, NPR1 has a unique role in the SA–JA crosstalk in various plants. In contrast with Arabidopsis and tobacco (*Nicotiana tabacum*), *NPR1* negatively regulates signal crosstalk, thereby preventing SA from suppressing JA accumulation during herbivory, which means that plants prefer producing JA during herbivory. Authors of [20,21,22] revealed that SA and not JA signaling plays a critical role in host resistance to pathogen infection, during which, rapid changes occur in the host plant to generate ROS, such as hydrogen peroxide (H_2_O_2_), which activates the SA and JA crosstalk as a defense response [23]. To maintain cellular redox homeostasis and to avoid the deleterious effects of ROS, plants activate antioxidant proteins, including superoxide dismutase, peroxidase, and catalase [24]. In most plants, SA is critical in the induction of PR genes, stomatal closure, and programmed cell death (PCD) in response to pathogen infection. The SA level does not increase upon inoculation with both fungal and bacterial pathogens in rice plants [25]; however, during antimicrobial defense, SA accumulates as a signaling molecule [26]. Yang et al. [27] proposed that SA biosynthesis in rice plants modulates the redox balance and protects against the oxidative stress caused by *Magnaporthe grisea*. Little is known about the effect of BLB stress on flavonoid accumulation and related gene expression, the SA–JA crosstalk and interaction with flavonoids, and pathogen responsive gene expression. The aim of our study was to evaluate the role of F3H overexpression in response to BLB stress and to elucidate a molecular cascade by which *F3H* overexpression alters plant tolerance toward BLB stress via the orchestrated activation of plant hormones, PSM, and PR genes in rice.

## 2. Materials and Methods

### 2.1. Plant Material, Growth Conditions, and Phenotypic Evaluation

We used the Nagdong F3H overexpressor (OxF3H) line and Nagdong wildtype (WT) rice seeds. Seeds were pre-treated with fungicides overnight, washed three times with double-distilled water, soaked for 3 days in the dark at 32 °C, with water being changed every day as described in [28]. After breaking the dormancy during soaking, the seeds were germinated in autoclaved soil and kept in the dark for 3 days. After the successful growth of most seeds, the seedlings were exposed to light and kept in a greenhouse for further experimentation. Phenotypic data of transgenic (OxF3H) and non-transgenic (WT) lines were randomly collected. Root–shoot length was calculated after 4 weeks of growth and the panicle length, tillering number, seed number/panicle, seed length, and seed weight were calculated when the plants were fully matured.

### 2.2. Experimental Design

We selected 3 groups of Nagdong to demonstrate the effect of BLB on rice plants. The 1st group was selected as the control (cont), which was not inoculated with Xanthomonas oryzae pv. oryzae. The 2nd and 3rd groups included wildtype- (WT-T) and overexpressor F3H-treated (OxF3H-T), respectively, which were both inoculated with the Xanthomonas oryzae pv. oryzae. We selected the K3a strain of Xanthomonas oryzae pv. oryzae as it causes BLB disease. K3a was used for inoculation after 6 weeks of seed germination, through the leaf clipping method [29], selecting the 3rd, 5th, and 7th leaves from WT-T and OxF3H-T plants. After inoculation, samples were collected in triplicates from random plants for RNA extraction, hormone analysis, and antioxidant activity after 0, 3, 6, 12, 24, and 36 h, placed in liquid nitrogen, and stored at −80 °C for further experimentation. As leaf lesion length differentiation started after 2 days, lesion length was measured in triplicates after 2, 3, 4, 5, and 6 days. Leaves for the in situ detection and quantification of flavonoids were collected after 3, 6, 12, and 24 h in triplicate after inoculation. However, protein expression was detected in only WT-T and OxF3H-T plants, 3, 6, and 12 h after inoculation in triplicate.

### 2.3. RNA Isolation and qRT-PCR

RNA extraction, cDNA synthesis, and qRT-PCR were performed as described in [28] to quantify the relative expression of F3H, FLS, DFR, SLR1, NPR1, and Xa1 genes. Briefly, total RNA was isolated using the RNeasy Plant Mini Kit (50) (Qiagen, Hilden Düsseldorf, Germany). The cDNA was synthesized using the cDNA synthesis kit qPCRBIO, and qRT-PCR was performed using the qPCRBIO SYBR Green kit (PCR Biosystems, Pennsylvania, USA), using actin as the housekeeping gene. PCR was performed using Eco Real-Time (Illumina, Singapore) and the total volume of reaction was adjusted to 20 µL, containing 10 µL SYBR green, 7 µL ddH_2_O, 1 µL template DNA, and 1 µL primer. The list of primers and accession number of each gene are listed in Supplementary Table S1. Each reaction was repeated three times.

### 2.4. Flavonoid In Situ Detection and Isolation

After the Xanthomonas oryzae pv. oryzae inoculation, fresh leaves collected after each time point were subjected to diphenylboric acid-2-aminoethyl ester (DPBA) staining. DPBA staining solutions were prepared by mixing 0.25 g (0.25%) DPBA and 200 µL Triton X-100 (0.02% *v*/*v*) in ddH_2_O up to a final volume of 100 mL. The leaf samples were then incubated in 0.25% staining solution in a vacuum for 5 min. After staining, the samples were mounted on microscope slides for confocal microscopy. A confocal laser scanning microscope (CLSM) (Carl Zeiss LSM700, Oberkochen, Germany) was used to detect the fluorescence of flavonoids. An FITC filter (suppression LP 488 nm) was used for visualizing Kr (green), while an R-PE filter (suppression LP 488 nm) and rhodamine (suppression LP 555 nm) were used for visualizing Qu (orange) and naringenin (red), respectively.

To quantify the flavonoid accumulation in response to BLB stress, we isolated Kr and Qu from the samples as described in [28]. Approximately 3 g of frozen leaves were ground in liquid nitrogen into a fine powder; homogenized in 30 mL methanol, water, and HCl mixture with the ratio of MeOH 79 mL, H_2_O 20 mL, HCl 1 mL; and shaken for 6 h. The crude extracts were filtered, the filtrates were diluted using a rotary evaporator to 2 mL at 30 °C, and further dried in a heating block at 60 °C overnight. The dried crude extract was dissolved in 1 mL HPLC grade ethanol. Reference standards for spectrophotometry were prepared by dissolving 1 mg of each standard sample in 1 mL ethanol. All samples were analyzed in triplicate.

### 2.5. Quantification of Endogenous SA and JA

To determine the crosstalk between SA and JA in response to BLB stress, we quantified both hormones. Leaves from the control, WT-T, and OxF3H-T plants were collected after 0, 3, 6, 12, and 24 h, and freeze-dried samples were ground in liquid nitrogen into a fine powder. The powder (0.3 g) was homogenized with 90% ethanol and 100% methanol and centrifuged for 20 min at 1000 rpm. The supernatant was collected, and methanol of the supernatant was dried in a vacuum centrifuge and resuspended in 5% trichloroacetic acid (3 mL). The supernatant was further mixed with ethyl acetate/cyclopentane/isopropanol (49.5:49.5:1, *v*/*v*) and the uppermost organic layer was collected in a 4 mL vial and dried with nitrogen gas. The extracted SA was analyzed by injecting a 1 µL sample into HPLC, with quantification via fluorescence detection. For the JA analysis, 0.2 g freeze-dried leaves were homogenized with liquid nitrogen and JA was extracted with acetone and 50 mM citric acid (70:30, *v*/*v*), as described in [28]. The internal standard [9,10-2H2]-9,10-dihydro-JA (20 ng) was added to the suspension. The extract was kept at a low temperature overnight to facilitate evaporation of highly volatile organic solvents and retain less volatile fatty acids. The remaining solution was filtered and extracted with 10 mL diethyl ether three times. The extract was loaded onto a solid-phase extraction cartridge (500 mg of sorbent, aminopropyl) and the cartridges were cleaned with 7.0 mL 2-propanol and trichloromethane (1:2, *v*/*v*). JA and standards were eluted with 10 mL diethyl ether and acetic acid (98:2, *v*/*v*). The residue of solvents after evaporation was esterified with diazomethane and analyzed by injecting a 1 µL sample into a GS-MS (6890N network GC system and the 5973-network mass-selective detector (Agilent Technologies, Palo Alto, CA, USA) in the selected ion mode. The ion fragment was monitored at *m*/*z* = 83 amu, consistent with the base peaks of JA and [9,10-2H2]-9,10-dihydro-JA; JA was quantified using the peak areas corresponding to the respective standards.

### 2.6. Protein Extraction and Immunoblot Analysis

To quantify the relative protein expression in WT-T and OxF3H-T plants, protein extraction and immunoblot analysis were performed, as described in [30]. Proteins from WT-T and OxF3H-T plants were collected at 3 time points (3, 6, and 12 h) after Xanthomonas oryzae pv. oryzae inoculation was extracted using 10 mL TCA/acetone (10% trichloroacetic acid [TCA] and 0.07% β-ME in acetone P.A.), as described in [31]. Equal amounts of protein were preboiled for 5 min, separated using 10% SDS-PAGE at 100 V for 150 min, and electroblotted onto a nitrocellulose (NC) membrane (Whatman, Tokyo, Japan) using the semi-dry method, running at 19 V for 90 min on a Trans-Blot DS semi-dry transfer cell (Bio-Rad). The NC membranes were blocked in TBST (0.1% Tween-20 in TBS) and 5% nonfat dry milk (*w*/*v*) for 2 h at room temperature. The membranes were incubated with primary rabbit anti-F3H antibodies in 5% nonfat dry milk (*w*/*v*) in TBST overnight at 4 °C, rinsed 3 times for 10 min each in TBST, incubated with Gt anti-Ms IgG (H + L) secondary antibody (Invitrogen, Massachusetts, USA) at a dilution of 1:1000 for 2 h at room temperature, and rinsed 3 times for 10 min each in TBST. The blot was developed using Amersham ECL (GE Healthcare, England, U.K.) and protein bands were exposed on an X-ray film.

### 2.7. GPx Activity and Measurement of MDA Contents

The activities of the antioxidant enzymes glutathione peroxidase (GPX) and malondialdehyde (MDA) were determined using the Glutathione Peroxidase Cellular Activity Assay Kit (Sigma, Saint Louis, USA) and Lipid Peroxidation (MDA) Assay Kit (Sigma), respectively, according to the manufacturer’s protocol. For the GPX detection, 100 mg deep-frozen leaves were ground in liquid nitrogen, homogenized in 3 mL 5% trichloroacetic acid (TCA), and centrifuged at 15,000 rpm for 15 min, as described in [32]. The extract was used for further analysis and blank, positive control, and sample reactions were performed according to the scheme presented in Table 1. The kit provided the glutathione peroxidase assay buffer, NADPH assay reagent, and Luperox 70% tert-butyl-hydroperoxide (TBH70X). According to the user manual, 1 vial of NADPH assay reagent was reconstituted in 1.25 mL ddH_2_O, which was enough for 20 tests. To prepare 30 mM tert-butyl-hydroperoxide, 21.5 µL Luperox TBH70X was diluted in ddH_2_O to 5 mL. All reactions were prepared in 1 mL volume, as described in Table 1, and then 250 µL was placed in a 96-well microplate. The reaction was initiated by adding 10 µL 30 mM tert-butyl hydroperoxide. The decrease in absorbance at 340 nm was calculated using wavelength 340 nm, initial delay 15 s, interval 10 s, and number of readings 6. The GPX activity was calculated in units/milliliter using the following formula:

ΔA_340_/6.22 × DF/V (1)

where

ΔA_340_ = A_340_/min_(blank)_ − A_340_/min_(sample)_;
6.22 = Ɛ^mM^ for NADPH;
DF = dilution factor of sample before adding to reaction;
V = sample volume in mL.

**Table 1:** Glutathione peroxidase activity reaction scheme.

	GPX Assay Buffer (μL)	NADPH Assay Reagent (μL)	Enzyme (0.25 unit/mL) (μL)	Sample (μL)	30 mM t-Bu-OOH (μL)
Blink	940	50	---	---	10
Positive control	900	50	50	---	10
Sample	900	50	---	50	10

For the MDA content measurements, the kit provided the MDA lysis buffer, phosphotungstic acid, BHT 100X, TBA, and 4.17 M MDA standard. The TBA solution was reconstituted by adding 7.5 mL glacial acetic acid (not provided), the volume was adjusted to 25 mL by adding ddH_2_O, and the solution was sonicated. To prepare 2 mM standard MDA, 10 µL 4.17 M MDA solution was diluted with 407 µL ddH_2_O and then 100 µL diluted solution was added to 900 µL ddH_2_O to prepare the 0.2 mM MDA solution. Subsequently, 0, 2, 4, 6, 8, and 10 µL 0.2 mM MDA standard solution was added into a 96-well microplate and 0 (blank) 0.4, 0.8, 1.2, 1.6, and 2.0 µL standards were prepared. Thereafter, ddH_2_O was added into each tube to reach a volume of 200 µL. Samples were prepared by homogenizing 10 mg tissue with 300 µL MDA lysis buffer containing 3 µL BHT on ice. The samples were centrifuged for 10 min at 13,000 rpm and the debris was discarded. Then, 200 µL of each sample was placed in a 1 mL tube, to which 600 µL TBA was added, incubated for 1 h at 95 °C, and cooled by keeping it on ice for 10 min. Finally, 200 µL blanks and samples were pipetted into a 96-well microplate and the absorbance was analyzed at 532 nm. The reaction was run in three technical replicates, the MDA contents were calculated in micromoles/gram, and data were calculated using the following formula;

S_a_/S_v_ × D = C (2)

S_a_ = amount of MDA in unknown sample (nmole);
S_v_ = sample volume added into each well (mL);
D = sample dilution factor;
C = concentration of MDA in the sample.

### 2.8. Amino Acid Isolation and Chlorophyll Content

Aspartic acid, proline, arginine, and total amino acids were quantified as described [33]. A total of 1 g of deep-frozen sample was ground into a fine powder in liquid nitrogen and extracted with 20 mL 70% HPLC grade methanol with shaking for 24 h. The amino acids were evaluated using the EZ:faast analysis kit (Phenomenex, Santa Clara, CA, USA). Amino acid content was determined via GC-MS using the Hewlett-Packard (HP) 6890N/5975 instrument (Agilent Technologies, Torrance, CA, USA) and a ZB-AAA (10 m × 0.25 mm) amino acid analysis column, with a constant carrier gas flow and an oven temperature program as described in [34]. The chlorophyll contents were measured via SPAD using randomly selected leaves in triplicate after the 1st, 2nd, 3rd, 4th, and 5th DPI.

### 2.9. Statistical Analysis

All experiments were performed in triplicate and the data from each replicate were pooled. Data were analyzed using two-way ANOVA, followed by the Bonferroni post hoc test (significant difference: *p* ˂ 0.001). A completely randomized design was used to compare the mean values of different treatments. Data were graphically presented and statistical analyses were performed using the GraphPad Prism software (version 5.01, GraphPad, San Diego, CA, USA).

## 3. Results

### 3.1. Overexpression of F3H Altered the Phenotypic Traits of Rice

**Figure 1:**
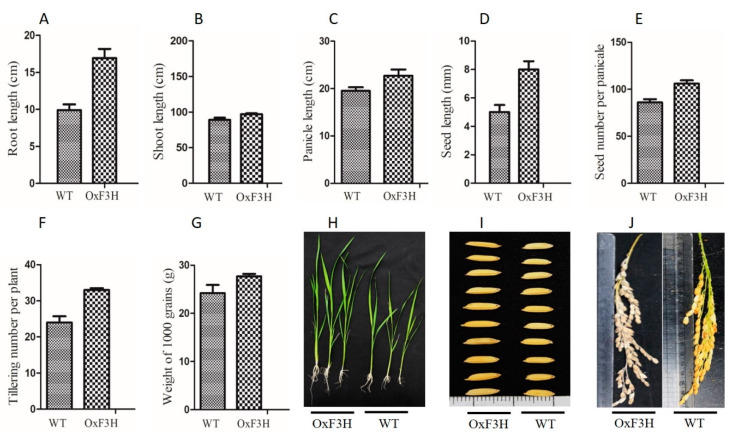
Evaluation of growth parameters of OxF3H and WT plants. (**A**) Root length, (**B**) shoot length, (**C**) panicle length, (**D**) seed length, (**E**) seed number per panicle, (**F**) tillering number per plant, (**G**) weight of 1000 grains, (**H**) pictorial representation of OxH3F and WT root/shoot length, and (**I**,**J**) pictorial representation of OxF3H and WT seed length and panicle length, respectively.

Transgenic lines overexpressing OxF3H were generated and verified in our previous study [28]. To evaluate the effect of F3H on the plant phenotype, we compared the transgenic and WT lines. Root, shoot, panicle, and seed lengths; seed number per panicle; tillering number; and weight of 1000 grains were significantly higher in the transgenic line (Figure 1). Root length increased by 20% in the OxF3H line. Similarly, shoot length increased by 8.9%, panicle length 15%, seed length 60%, seed number per panicle 23%, tillering number 37.5%, and weight of 1000 grains 12.5% in the OxF3H line. Thus, consistent expression of F3H regulated the secondary metabolites, which increased the phenotypic traits in rice.

### 3.2. Transgenic Rice OxF3H Showed Increased Tolerance to BLB

**Figure 2:**
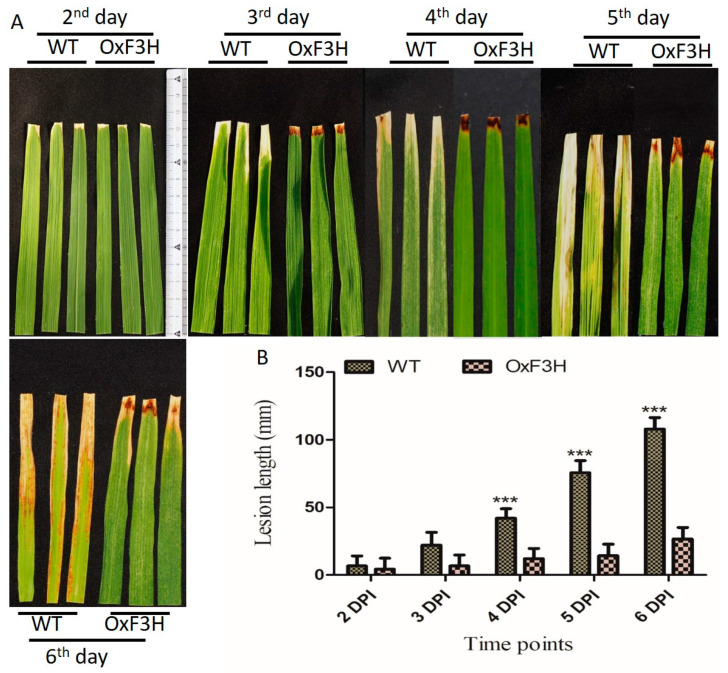
Identification of bacterial leaf blight infection leaf lesion length between OxF3H and WT line. (**A**) Pictorial representation and (**B**) quantitative representation of lesion lengths. Bars indicate mean ± standard deviation and *** show a significant difference (*p* ≤ 0.001), as analyzed using two-way ANOVA and a Bonferroni posttest. Data were collected after the second day of BLB infection every day until the sixth DPI.

To investigate the function of OxF3H in the plant’s defense, the tolerance level of the transgenic line to BLB-mediated stress was evaluated and compared with that of the WT plant. Lesion lengths in OxF3H and WT plants were measured 2, 3, 4, 5, and 6 days post-inoculation (DPI) using the leaf clipping method [35]. Quantitative measurement of the lesion length was calculated in millimeters; 53.8%, 230%, 250%, 435.7%, and 306% increases in lesion lengths in the WT compared with OxF3H was observed 2, 3, 4, 5, and 6 DPI, respectively (Figure 2A). Leaf lesion length in WT was significantly increased after the 4th, 5th, and 6th DPI (Figure 2B). Thus, the overexpression of OxF3H in the transgenic line increased the tolerance to BLB stress.

### 3.3. Cell Death and Antioxidant Regulation in Response to BLB Stress

**Figure 3:**
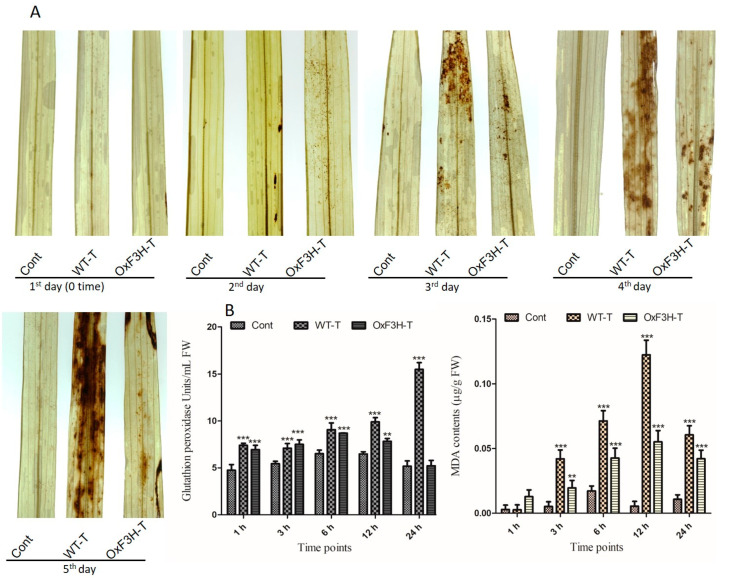
H_2_O_2_ detection with DAB staining and antioxidant accumulation in leaf segments of the control, OxF3H, and WT plants in response to BLB stress. (**A**) Histochemical detection of H_2_O_2_ after each DPI until 5 days. (**B**) Quantitative accumulation of antioxidants; the right graph shows MDA contents and the left graph shows glutathione peroxidase (GPX) activity. Bars indicate mean ± standard deviation and ** shows *p* ≤ 0.01) and *** shows *p* ≤ 0.001 significant difference, as analyzed using two-way ANOVA and a Bonferroni posttest.

To further investigate the role of OxF3H in the tolerance to BLB stress, we evaluated the hypersensitive response (HR) by measuring H_2_O_2_ in OxF3H-T, WT-T, and control leaves after pathogen post-inoculation using diaminobenzidine (DAB) staining (Figure 3A). At 1 DPI, all three types of leaves displayed no H_2_O_2_ accumulation. However, the WT-T leaves displayed higher H_2_O_2_ accumulation after 3 DPI compared with OxF3H-T leaves. Moreover, H_2_O_2_ visibly increased in the OxF3H-T leaves after 4 and 5 DPI. Quantitative image analysis revealed increased accumulation of H_2_O_2_ in WT-T compared with the OxF3H-T leaves. Thus, flavonoids were involved in mitigating the HR response. Plants have an enzymatic ROS scavenging mechanism for self-defense, which is rapidly activated during ROS generation. We determined the glutathione peroxidase (GPX) activity and malondialdehyde (MDA) contents in infected leaves (Figure 3B). GPX reduces peroxides into alcohols using glutathione to inhibit the formation of free radicals, which damage the cells. MDA accumulation reflects lipid peroxidation and cell membrane damage. We observed a consistent and significant (*p* ≤ 0.001) increase in GPX and MDA contents in both the OxF3H-T and WT-T plants compared with the control plants. Both GPX and MDA showed high accumulation in WT-T compared with the OxF3H-T plants. Initially, after 3 h, GPX was higher in OxF3H-T plants, but upon increasing stress, GPX increased in the WT-T plants compared with the OxF3H plants. Similarly, the MDA content was higher at 1 h, but after 3 h, it increased in WT-T plants as compared with the WT-T plants. Thus, reduced levels of GPX and MDA in the transgenic line were associated with the overexpression of *F3H*, which was involved in the mitigation of BLB stress.

### 3.4. BLB Stress Increased Flavonoid Accumulation

**Figure 4:**
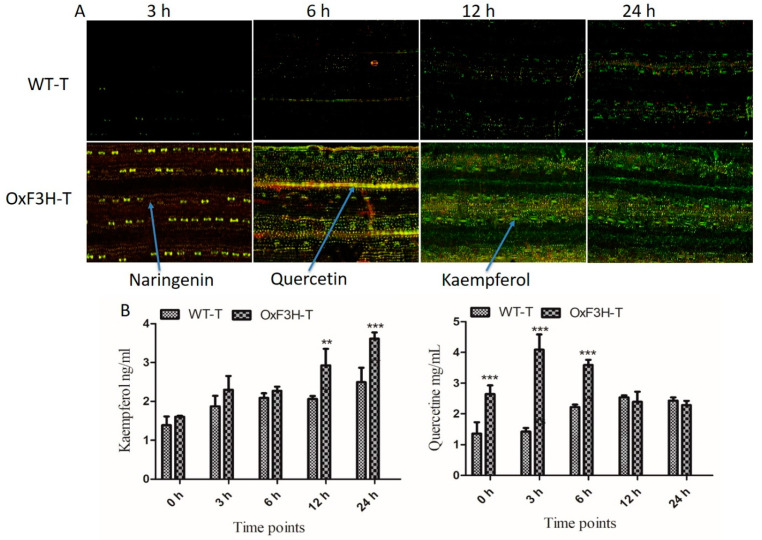
Detection and quantification of flavonoids in OxF3H and WT in response to BLB stress. Naringenin, kaempferol, and quercetin were visualized in plant tissue via DAPB staining using a confocal laser scanning microscope (CLSM). (**A**) Flavonoids weredetected in both the OxF3H and WT plants tissues, while arrows indicate different colors, where red shows naringenin, orange shows quercetin, and green shows kaempferol. Samples for in situ detection of flavonoids were collected after 3, 6, 12, and 24 h post-inoculation of Xanthomonas oryzae pv. oryzae. (**B**) Quantitative accumulation of flavonoids; the right graph shows quercetin while the left graph shows kaempferol accumulation in response to BLB stress at various timepoints. Graph bars indicate mean ± standard deviation and ** shows *p* ≤ 0.01 and *** shows *p* ≤ 0.001 significant difference, as analyzed using two-way ANOVA and a Bonferroni posttest.

F3H is a key regulatory enzyme of the flavonoid biosynthesis pathway and is activated during biotic stress. To investigate whether F3H expression increased the accumulation of flavonoids in plants during BLB stress, we evaluated Kr and Qu in WT-T and OxF3H-T plants after 3, 6, 12, and 24 h post-inoculation using diphenylboric acid-2-aminoethyl ester (DPBA) staining. Prominent accumulation of Kr and a small accumulation of Qu and naringenin were detected in WT-T plants after 6, 12, and 24 h post-inoculation (Figure 4A). Green, yellow, and red indicate KR, Qu, and naringenin, respectively (indicated by arrows). In the OxF3H-T line, after 3 h post-inoculation, a high concentration of naringenin was detected, followed by Qu, whereas Kr was absent in the initial stage. After 6 h, all three metabolites were visually detected at similar concentrations. In the later stages (12 and 24 h), a high concentration of Kr and a small amount of Qu were detected. Quantitative analysis of Kr and Qu in both the WT-T and OxF3H-T plants under pathogenic stress (Figure 4B) revealed that Kr increased consistently in both WT-T and OxF3H-T plants; however, the Kr concentration was higher in the OxF3H-T plants compared with the WT-T plants. Kr increased significantly (*p* ≤ 0.001) in the OxF3H-T plants after 12 and 24 h. The increased percentage of OxF3H-T compared with WT-T was 42.11% and 45.18% after 12 and 24 h, respectively. By contrast, Qu was initially highly accumulated in OxF3H-T plants, but after 6 h, the Qu levels started declining; however, in the WT-T plants, Qu accumulation increased consistently. The Qu content was significantly (*p* ≤ 0.001) higher in OxF3H-T as compared with WT-T after 6 h post-inoculation. After 12 and 24 h, the Qu content reduced non-significantly in the OxF3H-T compared with the WT-T plants. Thus, pathogenic attack induced the expression and accumulation of Kr and Qu, and that Kr and Qu could mitigate the stress. Naringenin is an important precursor of flavonoids and can metabolize to Kr and Qu during stress. Because Qu levels were higher compared with Kr levels in the initial stage of infection and Kr accumulated to a higher concentration later, Kr was more specifically involved in the mitigation of pathogenic stress. Moreover, F3H was induced under stress and regulated the flavonoid content in cooperation with other genes.

### 3.5. Expression of F3H Induced the Expression of Flavonoid Biosynthesis Genes and DELLA Protein

**Figure 5:**
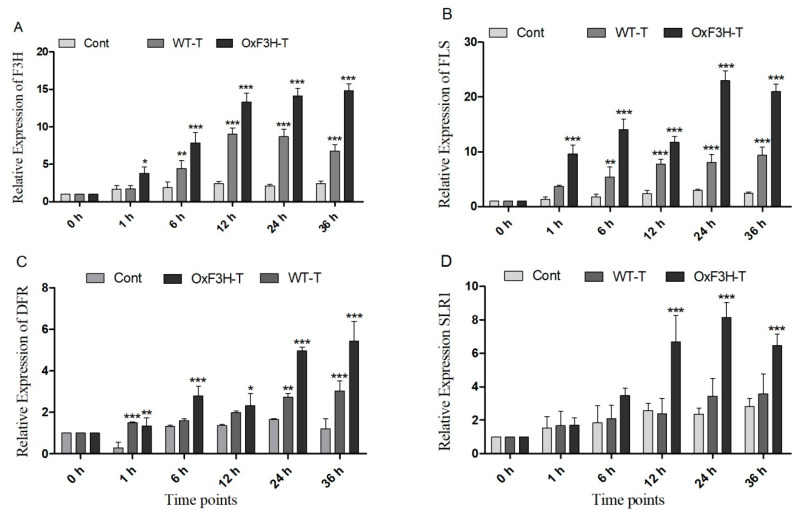
Relative expression patterns of F3H and related genes in the control, WT, and OxF3H plants. (**A**–**D**) F3H, FLS, DFR. and SLR1, respectively. The fold change of each gene was measured after each given time point and actin was used as the reference gene. Graph bars indicate mean ± standard deviation and * *p* ≤ 0.5, ** shows *p* ≤ 0.01 and *** shows *p* ≤ 0.001 significant difference, as analyzed using two-way ANOVA and a Bonferroni posttest.

In plants, the phenylpropanoid pathway generates several types of flavonoids by regulating various enzymes. Of these, F3H is a regulatory enzyme that metabolizes naringenin into dihydrokaempferol (DHK), which is further metabolized into various flavonoids [36]. We evaluated the induction of the genes downstream of F3H (FLS and DFR), which were responsible for the biosynthesis of different flavonoids in response to BLB stress in the control, WT-T, and OxF3H-T plants. F3H was constitutively expressed in OxF3H-T plants at different time points. The expression of F3H was higher in the OxF3H-T than in the WT-T plants, whereas F3H was significantly (*p* ≤ 0.001) expressed in both OxF3H-T and WT-T plants (Figure 5A). We evaluated protein expression in overexpressor and WT-untreated plants and found greater expression of F3H in the overexpressor line compared with the WT line (Supplementary Figure S1). Due to the consistent and significant expression of F3H in the WT-T plants, BLB stress could induce F3H. FLS and DFR are downstream and are responsible for the conversion of dihydrokaempferol and dihydroquercetin (DHQ) into kaempferol, quercetin, respectively, among other anthocyanins [37]. Both genes had a pattern of expression similar to F3H (Figure 5B,C). FLS expression was significantly higher in both pathogen-inoculated plants compared with the control plants, whereas the expression was higher in the OxF3H-T plants compared with the WT-T plants. Similarly, DFR was highly expressed in both the inoculated plants compared with the control. However, the expression in the WT-T plants was significant at 1, 24, and 36 h. Moreover, stress induced the expression of F3H, which regulates flavonoid biosynthesis through downstream genes. We previously reported that F3H expression increases anthocyanin biosynthesis, which induces the expression of SLR1 (DELLA protein), which further induces gerbilline (GA) synthesis [28,38]. Therefore, we investigated the relative expression of SLR1 in the control, WT-T, and OxF3H-T plants and found that SLR1 was highly and significantly upregulated 160% and 245% in OxF3H-T plants after 12 and 24 h of post-inoculation, respectively, and downregulated after 36 h (Figure 5D). Although the expression was high in WT-T as compared with the control plants, it was inconsistent and non-significant. Thus, the overexpression of F3H could significantly induce SLR1 through the increased biosynthesis of anthocyanins.

### 3.6. SA and JA Signaling and PR Gene Regulation in BLB Stress

**Figure 6:**
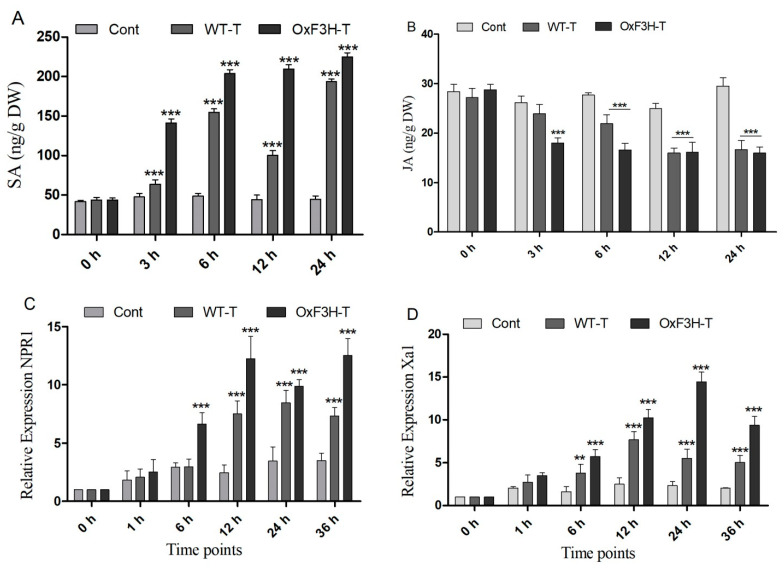
SA and JA signaling and PR gene expression. (**A**) SA and (**B**) JA accumulation in the control, WT, and OxF3H plants at different time points after Xanthomonas oryzae pv. Oryzae inoculation. (**C**,**D**) Relative expression of NPR1 and Xa1 genes, respectively, under BLB stress using actin as a reference gene. Bars indicate mean ± standard deviation and ** shows *p* ≤ 0.01 and *** shows *p* ≤ 0.001 significant difference, as analyzed using two-way ANOVA and a Bonferroni posttest.

Plants boost their immunity via the transcriptional modulation of defense genes through hormonally orchestrated signaling networks. Among various plant hormones, SA and JA are important components of the plant defense network and regulate the R genes in response to stress. Plants use the R genes encoded by nucleotide-binding site-leucine-rich repeats (NBS-LRRs), which recognize pathogen-released effector molecules, thereby inducing R-gene-mediated resistance [39]. In the current study, we investigated the induction of NPR1, Xa1, and SLR1 in association with SA and JA crosstalking (Figure 6A,B). We investigated the accumulation of SA and JA in the control, WT-T, and OxF3H-T plants 0, 3, 6, 12, and 24 h after inoculation. The results revealed that the SA accumulation consistently and significantly (*p* ≤ 0.001) increased in OxF3H-T plants compared with the controls (Figure 6A,B). Moreover, the accumulation of SA was significantly higher in the WT-T plants than the controls but lower than the OxF3H-T plants, indicating that F3H overexpression could increase SA accumulation. Unlike SA, JA was reduced in both the WT-T and OxF3H-T plants compared with the controls at all time points, except at 0 h, when the accumulation was non-significant (Figure 6A,B). JA was significantly (*p* ≤ 0.001) reduced after 6 (20%), 12 (33%), and 24 h (43%) in the WT-T plants and 40%, 35%, and 45%, respectively, in the OxF3H-T plants compared with the controls. However, the OxF3H-T plants showed more reduction than the WT-T plants. These results suggest that SA and JA antagonistically regulated the response to BLB stress. The relative expressions of the pathogen resistant genes NPR1 and Xa1 were evaluated 0, 1, 6, 12, 24, and 36 h after inoculation. Both genes showed the same pattern of expression. At 0 and 1 h, the expression was non-significant, but after 6 h onward, both genes were significantly (*p* ≤ 0.001) upregulated in the WT-T and OxF3H-T plants compared with the controls (Figure 6C,D). However, NPR1 expression was reduced by 148% at 24 h in the OxF3H-T plants and 109% at 36 h in the WT-T plants. The expression of Xa1 in the OxF3H-T plants reduced by 359% after 36 h and reduced 208% consistently in the WT-T plants after 12 h of inoculation. These results suggest that the overexpression of F3H was involved in the NPR1 and Xa1 induction, which was mediated by the SA–JA crosstalk.

### 3.7. Overexpression of F3H Increased Amino Acid and Chlorophyll Content under BLB Stress

The effect of BLB stress on the amino acid profile and chlorophyll content was investigated in the control, WT-T, and OxF3H-T plants. The results revealed that the content of aspartic acid, proline, and arginine and total amino acids increased in both the WT-T and OxF3H-T plants compared with the controls (Supplementary Figure 2A). However, the accumulation of amino acids was higher in the OxF3H-T plants than in the WT-T plants. The aspartic acid content increased significantly by 184.7% and 360.5% in the WT-T and OxF3H-T plants compared to control, respectively, whereas the OxF3H-T accumulation was 61% increased relative to the WT-T plants. Similarly, the proline content increased by 52.6% and 169.4% in the WT-T and OxF3H-T plants, respectively, compared with the controls. Arginine increased by 53.5% in the WT-T plants and 203.3% in the OxF3H-T plants compared with the controls, and the total amino acids increased by 63.9% and 189% in the WT-T and OxF3H-T pants, respectively, compared with the controls. The results confirmed that the overexpression of F3H increased the amino acid content and suggest that amino acids were upregulated upon pathogen attack. The chlorophyll content was consistently reduced in WT-T plants compared with the controls. In the OxF3H plants, the chlorophyll content increased non-significantly up to the third day of inoculation and thereafter significantly decreased (Supplementary Figure S2B). The significant reduction of chlorophyll occurred in both the WT-T and OxF3H-T plants after the fourth and fifth DPI (20.5% and 13% at the fourth DPI and 26% and 16.8% at the fifth DPI, respectively). The reduction in chlorophyll content was lower in the transgenic line than the WT line, which suggests that the transgenic line was more tolerant to BLB than the WT.

## 4. Discussion

Interest in the function of specialized metabolites in plants has increased due to their use in agronomy [40]. Metabolic regulation and transcriptional alteration are important aspects of a plant’s defense system and play a crucial part in understanding cellular responses during stress. Several physiological responses to stress are common in plants, including the transcriptional regulation of genes, activation of metabolic biosynthesis, and accumulation of antioxidants, which enable the cells to protect from environmental stresses. Experimental evidence to confirm the direct participation of flavonoids in stress mitigation [14] is lacking; therefore, we generated OxF3H transgenic lines, which overproduce flavonoids, and compared the rate of pathogen resistance in control, WT-T, and OxF3H-T plants. We focused on BLB because it is a devastating disease and greatly reduces the yield and quality of rice. Reports have shown that a mild BLB infection can reduce the yield by up to 20%, whereas severe infection reduces the yield by up to 50% [41]. The rate of infection of BLB depends on the strain, rice cultivar, geographical location, stage of plant growth, and seasonal variations [42].

To avoid the excessive use of antibiotics for protecting crops, the development of resistant transgenic lines is a more reliable and environmentally friendly strategy. Control, WT-T, and OxF3H-T plants are required to compare and validate the involvement of flavonoids’ BLB tolerance. Overexpression of F3H was shown to cause an overaccumulation of flavonoids and is critical in biotic and abiotic stress [43]. Our findings revealed that the constitutive overexpression of F3H increased plant growth and development. Agronomic traits, namely, root, shoot, panicle, and seed length; seed and tillering number; and weight of 1000 grains were much higher in the transgenic line compared with the WT line (Figure 1). The breakdown products of secondary metabolites are reused as plant growth regulators, and thus, add a second phase of growth defense patterns [44]. Compared with normal conditions, under stress, plants focus on stress tolerance instead of growth and development. Therefore, under normal conditions, F3H overexpressor plants have better agronomic traits compared with WT plants. In this study, the transgenic and WT plants were initially differentiated by measuring the agronomic traits, which is consistent with the overaccumulation of flavonoids and their impact on growth and development.

Being sessile in nature, plants have evolved disease tolerance strategies in response to pathogen attack through the activation of an indigenous defense system, which is controlled by multiple signaling pathways. In this study, F3H transgenic rice plants showed high levels of pathogen tolerance compared with WT plants, as seen by the leaf lesion length (Figure 2B) and DAB staining (Figure 3A). Flavonoids act as antioxidants and have free radical scavenging activity, which contributes to mitigating stress [14]. The glycosides of Kr and Qu, including kaempferol-3,7-dirhamnoside (KRR), kaempferol 3-O glucoside 7-O-rhamnoside (KGR), kaempferol 3-O-[6″-O-(rhamnosyl) glucoside] 7-O rhamnoside (KRGR), quercetin 3-O-glucoside 7-O-rhamnoside (QGR), quercetin 3-O-[6″-O-(rhamnosyl) glucoside] 7-O-rhamnoside (QRGR), and quercetin 3-O-rhamnoside 7-O-rhamnoside (QRR), are involved in the direct defense mechanism [9]. Flavonoid accumulation is involved in drought, salinity, UV light, and oxidative stress tolerance and reduces water loss during stress [45]. Additionally, these metabolites provide tolerance to pathogenic colonization in the host plant [46]. Our study suggested that BLB stress causes oxidative damage to host plant tissue in the form of ROS generation. ROS, including hydroxyl group (OH), H_2_O_2_, and superoxide anion (O^−2^), are generated in the cells due to the reduction of oxygen, which affects the plants on a daily and seasonal, basis. Consequences of ROS generation appear in the form of protein oxidation, DNA damage, and membrane peroxidation under both biotic and abiotic stress [47]. Plants mitigate the effects of ROS by regulating their antioxidant machinery using components that are enzymatic—including superoxide dismutase (SOD), catalase (CAT), GPX, and MDA—and non-enzymatic, including glutathione, carotenoids, ascorbic acid, and flavonoids [48]. Our results revealed that BLB stress-induced H_2_O_2_ generation and a high level of GPX and MDA were produced in WT-T plants, whereas the OxF3H-T plants mitigated the stress and reduced the accumulation of GPX and MDA better compared with the WT-T plants (Figure 3B). Thus, the WT plants were susceptible and OxF3H were tolerant to BLB stress. Furthermore, the induction of F3H increased the accumulation of Kr and Qu under stress, especially in OxF3H-T plants (Figure 4B).

Our findings revealed that Kr and Qu reduced oxidative stress by scavenging superoxides, consistent with other findings showing that Kr, Qu, and isoquercitrin are powerful superoxide-scavenging agents [49]. Notably, the flavonoid B ring at position 3 is the active site for superoxide scavenging. Naringenin, the precursor of Kr and Qu, is rapidly metabolized by the activation of F3H during stress. Initially, during stress, naringenin accumulation was higher than that of Kr and Qu; however, after 6, 12, and 24 h of infection, naringenin accumulation decreased and Kr and Qu accumulation increased (Figure 4A). Studies have shown that antioxidant flavonoids are generated in the mitochondria, chloroplast, and mesophyll cells and in response to severe oxidative damage caused by ROS [50]. We investigated whether both Kr and Qu had a high accumulation in stomatal cells because they may participate in stomata opening and closing. Flavanols (Kr and Qu) not only scavenge ROS but also inhibit ROS generation [50]. Studies reported that flavonols regulate plant cell growth and differentiation and protein kinase activity, which in turn activate ROS-induced signaling cascades that are vital for cell growth and differentiation [51,52].

Plants initiate the hormonal machinery in response to stress tolerance. SA is one of the main participants in stress tolerance. Our results revealed that SA was significantly induced by BLB stress in both the WT-T and OxF3H-T plants, but the accumulation was higher in the transgenic line, which indicates that the overexpression of F3H increases SA accumulation (Figure 6A). However, JA accumulation was consistently reduced in both the WT-T and OxF3H-T plants, which indicated that JA and SA regulate antagonistically. Most studies support the hypothesis that SA and JA interact antagonistically [53]. Both SA and JA are important molecules in plant defense responses; however, the contribution of both depends on the invading pathogen. SA plays a major part in basal resistance to bacterial infection, whereas JA responds to fungal pathogens [53]. In response to pathogenic stress, the activation of the SA- or JA-mediated signaling pathway usually accompanies the induction of pathogenesis-related genes. In our study, we evaluated the expression of NPR1 and Xa1, which are tolerant to BLB stress. NPR1 is a master transcriptional co-regulator of SA-dependent genes, which significantly regulates using various SA-dependent modifications in the SA signaling pathway [54]. It was reported that Xa1 belongs to the nucleotide-binding-site leucine-rich repeat (NBS-LRR) class of the plant disease-resistant gene that encodes a cytoplasmic protein [7]. Our study indicated that NPR1 and Xa1 genes were induced by BLB stress, which indicates that they are functionally involved in the mitigation of BLB stress (Figure 6C,D). Sometimes, SA takes transcriptional control over JA via the induction of negative regulators, such as WRKY TF, which can inhibit the expression of JA-responsive genes [55]. Furthermore, it is indicated from the model developed by [56] that H_2_O_2_ generated by stress triggers SA biosynthesis, which is critical for defense responses, such as stomatal closer and cell death.

We found that overexpression of F3H induced downstream genes, such as FLS, DFR, and SLR1 (Figure 5). FLS catalyzes the conversion of dihydrokaempferol (DHK) and dihydroquercetin (DHQ) into Kr and Qu, respectively, whereas DFR is involved in anthocyanin biosynthesis. Although we did not quantify anthocyanin biosynthesis, DFR expression in OxF3H-T indicates that during stress, anthocyanin synthesis is upregulated for maintaining plant growth. Interestingly, our findings indicated an important function of F3H-mediated anthocyanin synthesis under stress. The FLS-mediated increase in anthocyanin biosynthesis induced SLR1 expression, which was responsible for the expression of the growth repressor DELLA. However, the interaction between anthocyanin, DELLA, and GA in plant immunity is not understood. Studies in Arabidopsis and wheat have revealed that DELLA promotes resistance to necrotrophic and susceptibility to biotrophic infection by altering the SA–JA balance [57,58].

In addition to flavonoids, hormones, and antioxidants, amino acids are an essential component of the plant immune system. BLB mediated stress induced the production of aspartic acid, proline, arginine, and total amino acids more significantly in the OxF3H-T plants than the WT-T plants (Supplementary Figure S2A). The biosynthesis of these special amino acids and free amino acids occurs in various metabolic pathways under various types of stress. To cope with environmental stress, amino acids participate in synthesizing proteins, act as regulatory and signaling molecules, are active participants in energy-associated metabolites, and have important functions in growth and development and adaptive responses [59]. We hypothesize that amino acid accumulation in the overexpressor line helps to maintain normal growth and development under stress. Similarly, photosynthesis plays a critical role in plant physiology and its alteration has a key role in plant defense against biotic and abiotic stress. Chlorophyll contents maintain the activity of photosynthesis at the cell, leaf, and whole plant levels. We found that high chlorophyll content accumulated in the OxF3H-T plants compared with the WT-T plants, which indicated that chlorophyll content increased plant tolerance to stress, as reported in [60].

**Figure 7:**
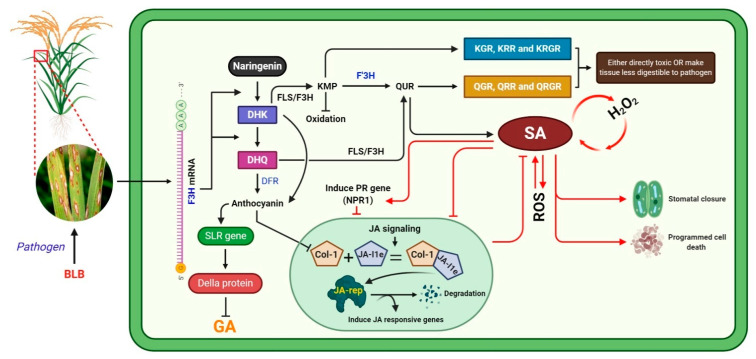
Proposed flow chart of the regulation of the plant defense system mediated by the F3H gene.

In conclusion, we evaluated the regulation of the rice defense system’s associated F3H overexpression in response to BLB caused by pathogenic Xanthomonas oryzae pv. Oryzae (Xoo) bacteria (Figure 7). BLB stress induces *F3H*, which is a key participant in the flavonoid biosynthesis pathway and can orchestrate a plant’s defense. Overexpression of *F3H* increases Kr, Qu, and anthocyanin production, alters SA–JA signaling, and regulates *SLR1*. The increase in the level of flavonoids, SA, NPR1, and DELLA protein increased BLB stress tolerance and maintained plant growth and development. Kr and Qu, which have strong antioxidant activities, could increase the SA accumulation. Further, SA induced the expression of PR genes and antagonistically reduced JA accumulation. We speculate that either the glucosides of Kr and Qu were toxic to Xanthomonas oryzae pv. oryzae bacteria or the accumulation of SA induced PCD and stomata closure. Furthermore, F3H regulated DFR, which regulates the biosynthesis of anthocyanin, which has a key role in maintaining plant growth and development during stress. Anthocyanin increased SLR1 expression, which suppresses DELLA expression and consequently reduces GA biosynthesis. Studies have reported that anthocyanin inhibits the JA pathway due to the suppression of Col-1 unit of conjugates of Col-1_+_ JA Ile, which is an inducer of JA responsive genes; however, we found that JA accumulation reduced under stress.

## Data Availability

The data that support the findings of this study are available on request from the corresponding author.

## Supplementary Materials

The following are available online at https://www.mdpi.com/article/10.3390/ijms22116152/s1, Figure S1: Western blot analysis of F3H protein accumulation in OxF3H and WT plants, Table S1: List of genes, primers and accession number.
