# Supplementary material for: Flavonone 3-hydroxylase Relieves Bacterial Leaf Blight Stress in Rice via Overaccumulation of Antioxidant Flavonoids and Induction of Defense Genes and Hormones
Source: Int J Mol Sci. 2021 Jun 7;22(11):6152. doi: 10.3390/ijms22116152 (PMC8201380; doi:10.3390/ijms22116152)
Supplement: Supplementary file 1 [file ijms-22-06152-s001.zip › ijms-1230732-supplementary.pdf]

Table S1. List of genes, primers and accession number.

S.No	Gene Name	Primer	Accession No
1	F3H	ATGGCGCCGGTGGCCACGAC (sense)<br>GATGAATCCGCCCTTCTTGCCG (antisense)	MT980835.1
2	FLS	ATGGCGGAGGTGCAGAGCGT (sense)<br>TTACATGGGGAGCTTATTGATCTTGCA (antisense)	XM_015769329.2
3	DFR	ATGGGCGAGGCGGTGAAGGG (sense)<br>TCATTTGACCAACGCTTCTGTTTCAGC (antisense)	MK636607.1
4	SLR1	ATGAAGCGCGAGTACCAAGAAGC (sense)<br>TCACGCCGCGGCGACGCGCCATGCC (antisense)	XM_015776057.2
5	NPR1	ATGGAGCCGCCGACCAGCCACGT (sense)<br>TCATCTCCTTGGTCGAATGGC (antisense)	HM991169.1
6	Xa1	ACTGCCCTCTTGCACACGCCTTTGG (sense)<br>CCGGTACATCAGTATTGTCCATCGG (antisense)	AB002266

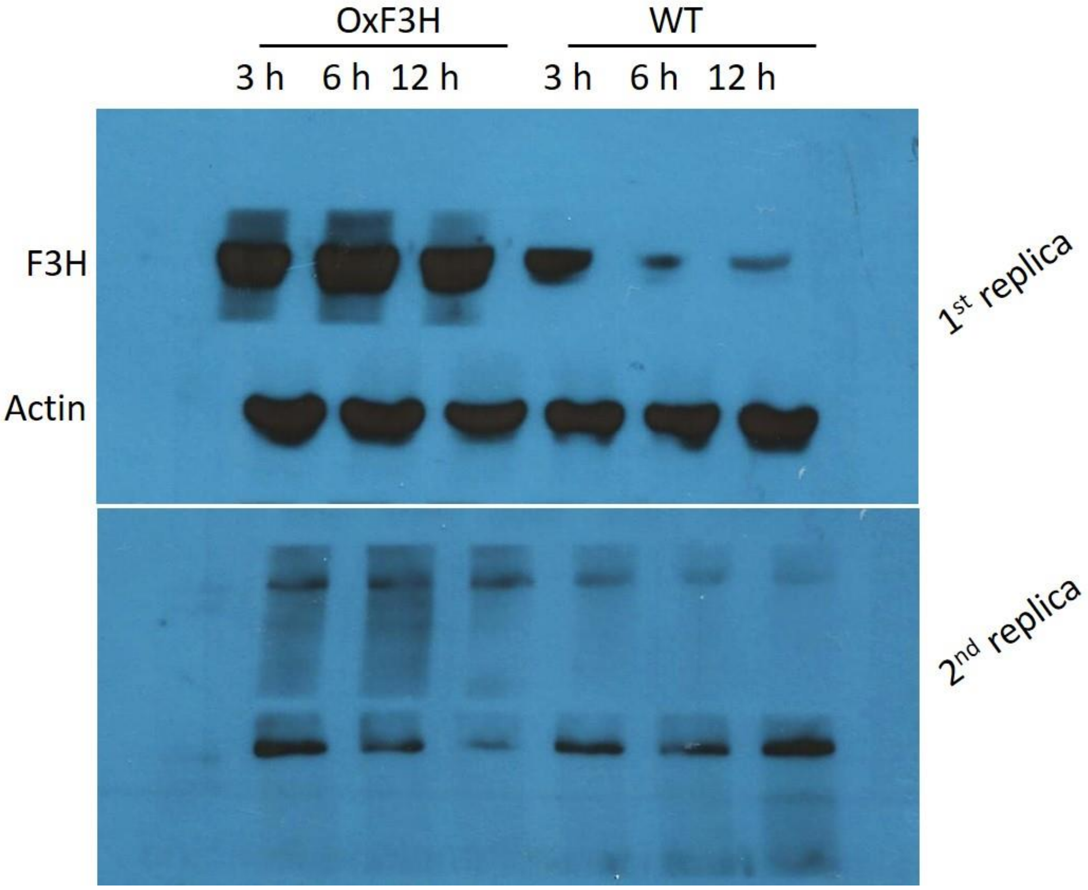

Figure S1. Western blot analysis of F3H protein accumulation in OxF3H and WT plants. The expression pattern was analyzed after 3, 6 and 12 hour of post inoculation of Xoo. Actin was used as reference protein.
